# Sea level rise and the drivers of daily water levels in the Sacramento-San Joaquin Delta

**DOI:** 10.1038/s41598-023-49204-z

**Published:** 2023-12-17

**Authors:** H. Baranes, S. L. Dykstra, D. A. Jay, S. A. Talke

**Affiliations:** 1 Climate Center, Gulf of Maine Research Institute, Portland, ME USA; 2 College of Fisheries and Ocean Science, University of Alaska Fairbanks, Fairbanks, AK USA; 3 School of the Earth, Ocean, and Environment, University of South Carolina, Columbia, SC USA; 4 Civil and Environmental Engineering Department, Portland State University, Portland, OR USA; 5 Civil and Environmental Engineering Department, California Polytechnic State University, San Luis Obispo, CA USA

**Keywords:** Hydrology, Physical oceanography

## Abstract

Water levels in deltas and estuaries vary on multiple timescales due to coastal, hydrologic, meteorologic, geologic, and anthropogenic factors. These diverse factors increase the uncertainty of, and may bias, relative sea level rise (RSLR) estimates. Here, we evaluate RSLR in San Francisco Bay and the Sacramento-San Joaquin Delta, USA by applying a physics-based, nonlinear regression to 50 tide gauges that determines the spatially varying controls on daily mean water level for water years 2004–2022. Results show that elevated river flow and pumping (99th percentile) raise water level up to 6 m and lower it up to 0.35 m, respectively, and coastal water level variations are attenuated by 30-60% within the Delta. Strong westerly winds raise water level up to 0.17 m, and tidal-fluvial interaction during spring tides and low discharge raises water level up to 0.15 m. Removal of these interfering factors greatly improves RSLR estimates, narrowing 95% confidence intervals by 89–99% and removing bias due to recent drought. Results show that RSLR is spatially heterogeneous, with rates ranging from − 2.8 to 12.9 mm y^-1^ (95% uncertainties < 1 mm y^-1^). RSLR also exceeds coastal SLR of 3.3 mm y^-1^ in San Francisco at 85% of stations. Thus, RSLR in the Delta is strongly influenced by local vertical land motion and will likely produce significantly different, location-dependent future flood risk trajectories.

## Introduction

Worldwide, sea-level rise (SLR) and water level variability affects major population, agricultural, and commercial centers in deltas or tidal rivers, with coastal effects extending hundreds of kilometers inland from the coast. Examples include the Sacramento-San Joaquin Delta, USA; Yangtze River, China; the Nile River Delta, Egypt; and the Mekong Delta, Vietnam. Major cities on tidal rivers include Shanghai, China; Hamburg, Germany; Rotterdam, Netherlands; Bangkok, Thailand; and London, United Kingdom. Nonetheless, relative sea level rise (RSLR) projections are typically only made at the coast^1–5^, and/or neglect the local hydrodynamic, hydrological, meteorological, anthropogenic, and geological factors that influence inland sea-levels.

Water levels and RSLR rates in deltas and rivers are influenced by coastal water level trends and variability, hydrologic changes, spatially varying vertical land motion (VLM), human modifications (e.g., dredging, structures), astronomical tidal variability, tidal evolution, and dynamical interactions between river flow and tides^6–13^. For example, RSLR in Shanghai has been strongly influenced by groundwater pumping and resulting land consolidation^14,15^. Other cities severely impacted by large and spatiotemporally variable land subsidence include Jakarta, Indonesia^16,17^; Tokyo, Japan^18^; and New Orleans, USA^19^. Furthermore, vulnerability to RSLR varies spatially along tidal rivers depending on which factors—coastal forcing, changing tides, river flow, VLM, and/or human modifications—dominate changes in both mean and extreme water levels^9,20^. A combination of multiple dynamical factors and high spatial variability leads to uncertainty and sometimes bias in estimates of long-term mean water level trends^21,22^.

**Figure 1:**
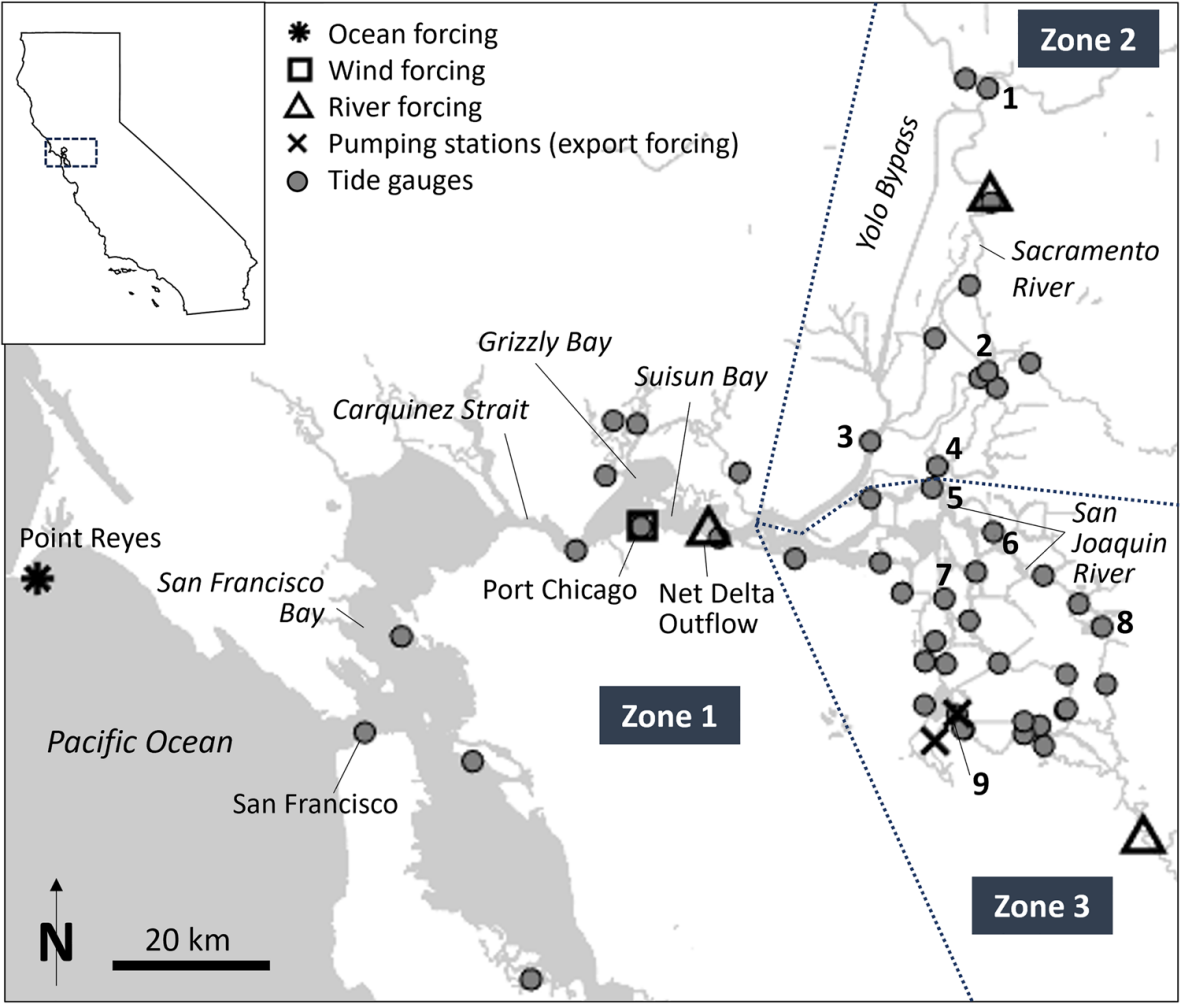
Map of the Sacramento-San Joaquin Delta and San Francisco Bay region showing tide gauges (gray circles) and locations of ocean, river, wind, and export forcing used as inputs into the regression model. Numbers denote locations/stations mentioned in the text (1 = Sacramento, 2 = Cross Delta Channel, 3 = Rio Vista, 4 = Georgiana Slough, 5 = San Andreas Landing, 6 = Venice Island, 7 = Bacon Island, 8 = Garwood Bridge/Stockton, 9 = Clifton Court Intake). Dashed lines delineate the three zones used in Eq. 1 (Methods): the bays and straits west of the Sacramento-San Joaquin Rivers confluence (zone 1), the northern Delta (zone 2), and the southern Delta (zone 3). The upper-left inset shows the location of the Delta within California. This map was created using the U.S. Census Bureau’s 2021 TIGER “AREAWATER” shapefiles.

Here, we evaluate the spatially varying controls on daily mean water level (DMWL; see Methods) and estimate RSLR rates over the past two decades in the Sacramento-San Joaquin Delta region (henceforth “the Delta”; Fig. 1), a ~ 3000 km^2^ area that encompasses both major cities (e.g., Sacramento & Stockton) and valuable farmland^23^. The system is an inverted delta that drains a 194,000 km^2^ watershed into San Francisco Bay through the Sacramento and San Joaquin Rivers (average flows of 550 and 100 m^3^/s, respectively) and multiple smaller rivers and creeks^24^. The Delta provides freshwater to 27 million people and 15,000 km^2^ of agricultural land^23^, much of which is delivered via state and federal water projects that pump up to 350 m^3^ s^-1^ of freshwater from the southwestern Delta^25,26^.

The Delta is also a transition zone between fluvial and oceanic influences. River flow dominates water level variations in parts of the eastern Delta^27^ but becomes less important in westerly regions. By contrast, the magnitude of tidal forcing and non-tidal sea level fluctuations generally decreases in the inland direction^27^. For example, the mean tidal range during low-flow conditions is 1.12 m at Port Chicago, but 0.28 m in Sacramento and 0.92 m in Stockton (data from the National Oceanographic and Atmospheric Administration (NOAA); see Fig. 1 for place names). As in other fluvial systems, tidal range is considerably reduced when river flow is elevated, especially along the Sacramento River upstream of Rio Vista^7,28–31^.

Understanding drivers of water level in the Delta is critical because hydrologic and coastal forcing (including SLR) drive flood risk and pose severe management challenges^23,32,33^. Subsidence compounds the increasing flood risk from coastal SLR in the 100+ islands in the Delta, with present-day subsidence rates estimated to vary from 3 to 20 mm yr^-1^. Much of the Delta region was formerly a tidal wetland, where peat accumulated as SLR occurred over the past 6700 years^34–37^. Delta islands began subsiding in the late-1800s and early-1900s when marshland was drained and leveed for agriculture^38^. Island subsidence is driven primarily by soil oxidation and secondarily by consolidation (e.g., groundwater withdrawals), and many islands lie below sea level, having subsided more than 5 m since the late 1800s^39–41^. Similarly, subsidence rates on the > 2100 km of Delta levees average 10–20 mm yr^-1^, with peak rates of up to 50 mm yr^-1^^42^. Available evidence suggests that island subsidence rates have decreased over the last century as the availability of highly organic soil has decreased^43^.

Changes in riverine and tidal forcing may also drive future shifts in flood risk. Instrumental measurements suggest that river discharge to San Francisco Bay has decreased by 30% since the 1800s, primarily during the spring season^44,45^. Nonetheless, model projections suggest that increased precipitation rates and a lower snow fraction may more than double runoff from extreme events reminiscent of the great 1862 flood in the next century, compounding the effects of SLR and subsidence^46^. Similarly, from the ocean, the greater diurnal tidal range at San Francisco increased by ~ 3.6% during the twentieth century^47^ to its current value of 1.78 m (see also ref.^12^). Any positive trends and variations in mean and extreme water levels increase seepage rates through levees^35,48^ and, in turn, increase the probability of levee overtopping or failure^43,49^.

Assessing controls on DMWL and isolating the influence of RSLR in the Delta requires an approach that simultaneously represents the influences of coastal and fluvial processes^50^. Here, we apply a physics-based non-linear regression to evaluate the spatially varying controls on DMWL and estimate RSLR rates at 50 gauges in the Delta region (Fig. 1) over water years 2004–2022 (October 1, 2003 to September 30, 2022). This time period was chosen due to the spatial coverage, quality, and datum control of available water level data relative to previous periods. Our local RSLR estimates are primarily influenced by coastal SLR and VLM over this relatively short time scale. Long-term secular trends in water level from tide gauge records include VLM, but they often record a minimum VLM rate, as they do not record subsidence in shallow strata^1,51^. Our RSLR estimates are probably not influenced much by hydrodynamic factors that evolve on secular timescales due to changes in system depth and geometry^9,52^. However, events such as a 2004 levee failure and installations of temporary drought barriers in 2015, 2021, and 2022 may influence water levels locally^53^. Wetland restoration or other water-infrastructure management may also cause long-term water level shifts in some areas. Undocumented datum shifts and uncharacteristically large water level perturbations are flagged by our quality assurance and lead to greater uncertainty bounds in estimated RSLR rates (see Methods).

Our approach modifies a non-linear regression developed by Refs.^52,54–56^ and based on a theory of tidal propagation in a convergent channel with friction and river flow. We regress DMWL at each gauge as a linear function of up to 6 independent physical processes (Eq. 1). Regression terms include river discharges, frictional tidal-fluvial interactions, non-tidal coastal sea level variation (due to coastal setup, for example, as measured at the Point Reyes tidal gauge), wind, exports (i.e., water diversions), and secular RSLR. A unique set of coefficients and exponents is calculated for each gauge via robust multiple linear regression. Values of regression terms both illuminate the spatially varying influences of physical forcing factors (i.e., river flow, tidal-fluvial interaction, coastal processes, wind, and exports) on DMWL, and enable statistically robust estimates of RSLR rates. Results indicate the approach is generally applicable to other deltas, tidal rivers, and coastally influenced inland locations.

## Results

### Regression model performance

**Figure 2:**
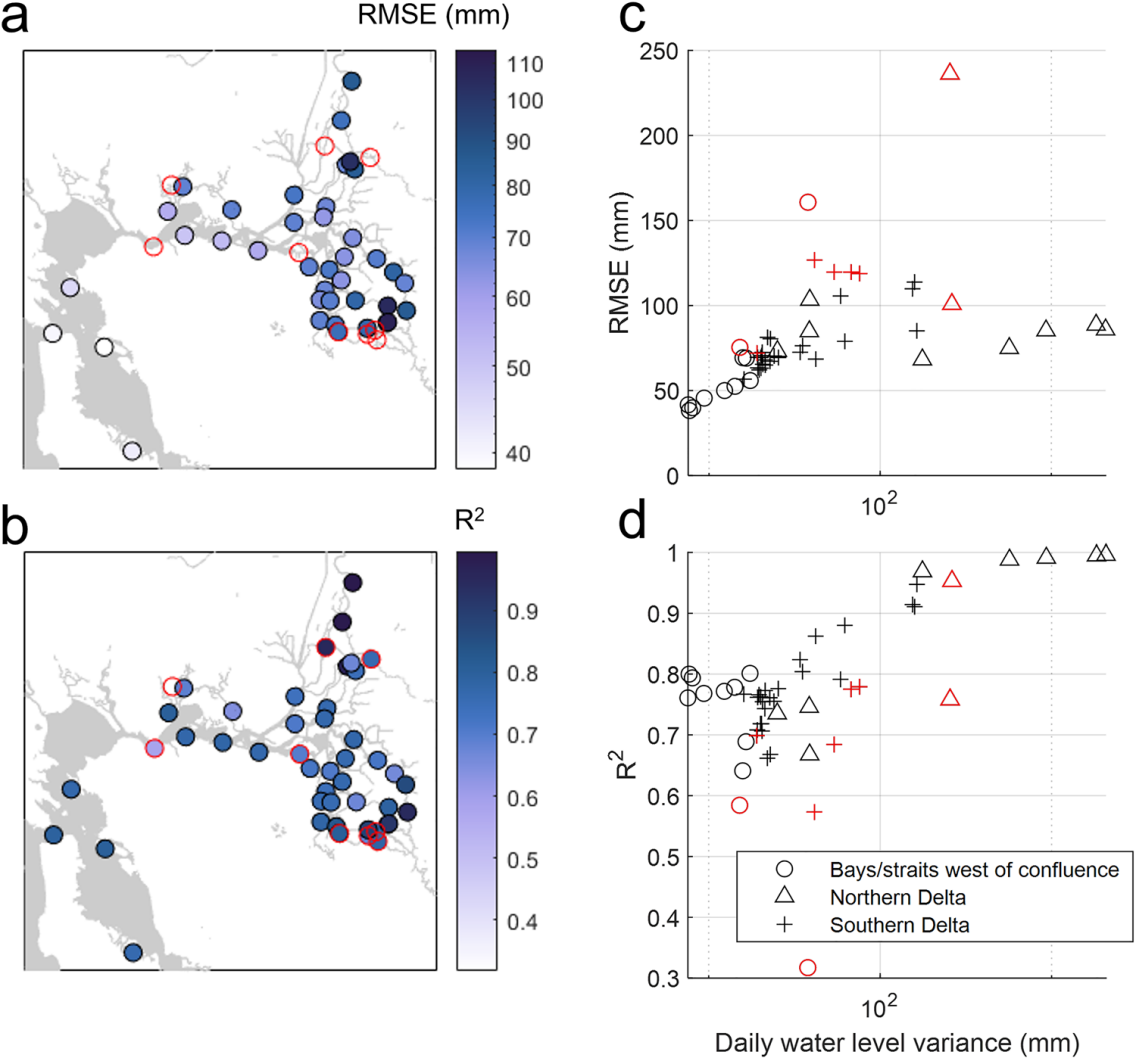
Regression performance. (**a**) Root mean square residual error (modeled minus measured daily mean water level). Stations where the regression did not perform well are outlined in red. Their large RMSE values are not included in the color map (see panel c) to preserve detail at lower values. (**b**) R^2^, or adjusted square error. (**c**, **d**) RMSE and R^2^ as a function of daily water level variance (i.e. the dynamic range in water level) at each station. Symbols delineate the geographic locations of stations, following zones in Fig. 1.

Our regression approach (see Methods) predicts DMWL well at most San Francisco Bay and Delta stations (Supplementary Table S1) and captures water level variations caused by fluvial and coastal forcing (Supplementary Fig. S4). Root-mean-square residual errors between modeled and observed DMWL are small (40–110 mm), and adjusted squared error (R^2^) varies between 0.67 and 0.99 at 41 out of 50 stations (Fig. 2a). Further, residual errors are uncorrelated with forcing parameters at these stations (for example, residual error does not change as a function of river discharge; see Supplementary Figs. S1–S3). We note that our analysis excludes days where discharge exceeded the 99th percentile because flow routing and other water resource management activities during high flow periods affected the model (see Methods). RMS error and R^2^ values generally increase upstream as river flow drives greater variance in DMWL (Fig. 2c). The lowest R^2^ values are generally found in seaward and lower-San Joaquin River stations where the dynamic range in daily mean water level is smallest, and noise and other processes not included in the regression represent a larger fraction of the variance (Fig. 2d).

**Figure 3:**
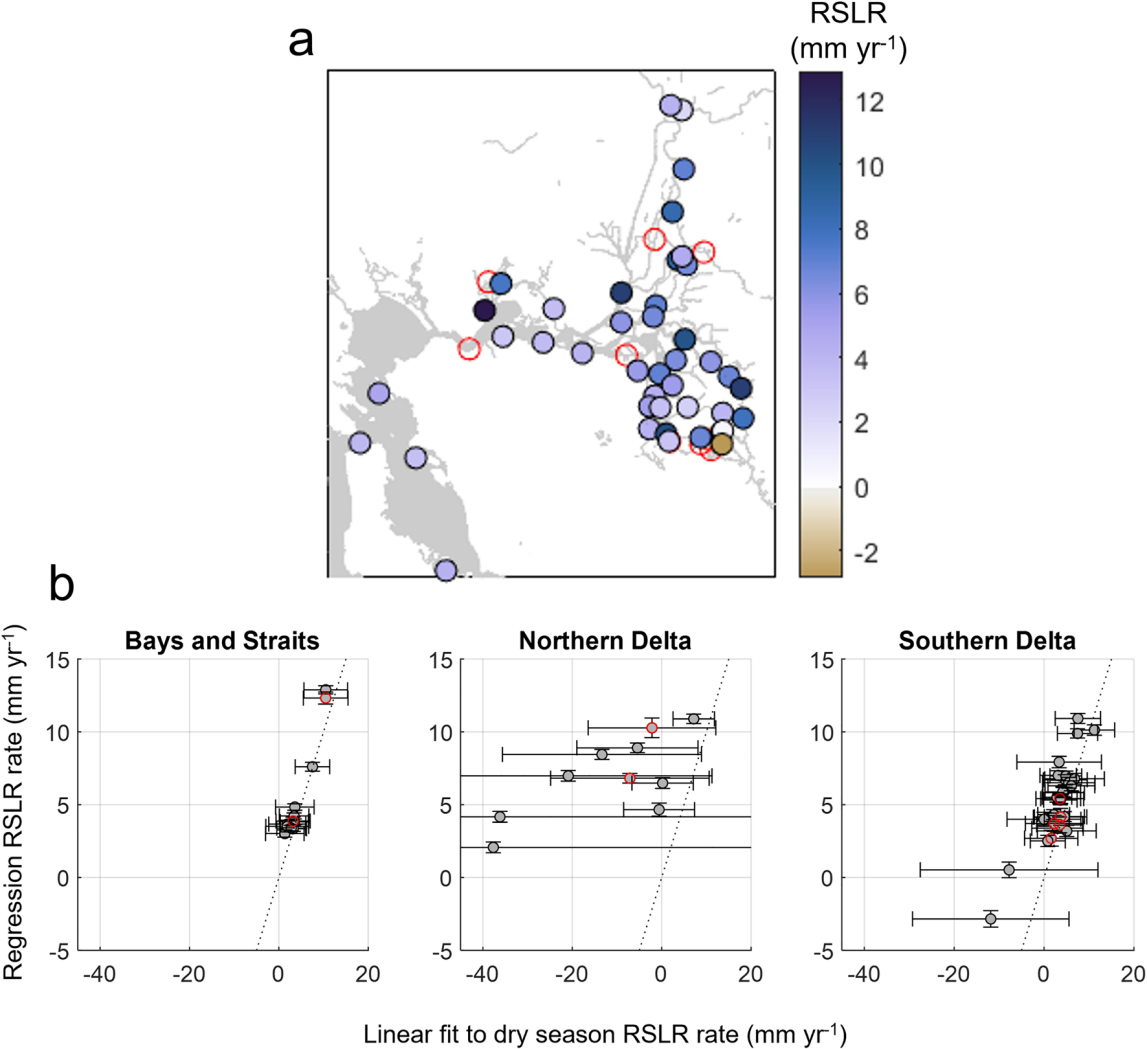
Relative sea-level rise rates. (**a**) Spatially-varying regression-based median estimates of RSLR (coefficient a_8_, Eq. 1; see Supplementary Table S1 for values with 95% confidence intervals). Stations where the regression did not perform well are outlined in red. (**b**) Regression RSLR rates versus the slope of a linear fit to dry season water levels. Vertical error bars show the 95% confidence interval for regression estimates, and horizontal error bars show the 95% confidence interval for the linear fit (values are listed in Supplementary Table S1).

**Figure 4:**
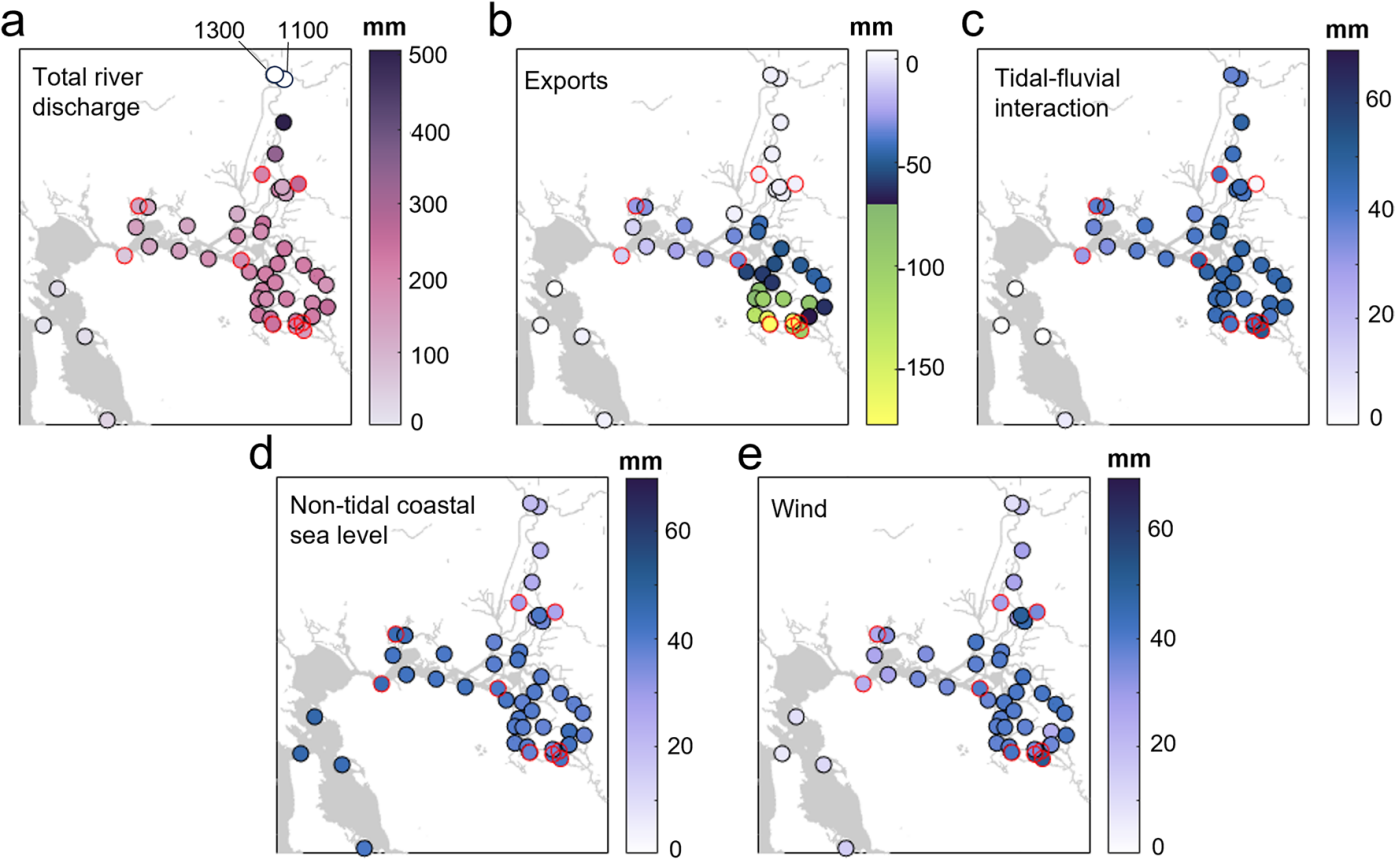
Spatially-varying contributions to daily mean water level. Contributions of each regression term are calculated using median forcing conditions (Table 1) and computed exponents and coefficients (Supplementary Table S1). Stations where the regression performed poorly are outlined in red. Note that we use a different color scale for discharge and exports to indicate greater forcing. (**a**) Total river discharge forcing, or the sum of the a_1_, a_2_, and a_3_ terms (Eq. 1). Large forcing values at the two northernmost stations (near Sacramento) are labeled and plotted in white to preserve detail on the color scale. (**b**) Export (pumping) forcing, or the a_4_ term. Note the reversed color axis. (**c**) Tidal-fluvial interaction forcing, or the a_5_ term. (**d**) Non-tidal coastal sea level forcing, or the a_6_ term. (**e**) Wind forcing, or the a_7_ term.

The poor performance of the model at some locations indicates that other factors besides coastal forcing, wind, and discharge from major rivers are sometimes influencing measured water levels. At 6 of the 9 stations where the regression performs poorly, large residuals occur during low river flow conditions (station 7 in Supplementary Fig. S1 and stations 24, 35, 41, 42, and 44 in Supplementary Fig. S3); these locations may be influenced by water diversions, tidal gates, construction of seasonal channel barriers, and other management activities^59^. At the remaining 3 poorly performing stations, residuals are uncorrelated with any forcing parameters (station 5 in Supplementary Fig. S1 and stations 16–17 in Supplementary Fig. S2). Residual errors at stations 5 and 17 suggest an undocumented datum shift. Elevated errors at station 16 may be due to its location near the confluence of two minor rivers not included in the regression. Regression coefficients, particularly RSLR estimates, may be unreliable at these 9 stations, so we do not analyze them further (these stations are also outlined in red Figs. 2, 3, 4 and starred in Supplementary Table S1).

### Relative sea level rise and physical drivers of water level

Results show that RSLR rates are highly variable and spatially heterogenous, with rates that range from − 2.8 to 12.9 mm yr^-1 ^(Fig. 3a; Supplementary Table S1). 95% confidence interval widths are relatively narrow, ranging 0.3 to 1.1 mm yr^-1^. At 30 of the 41 stations, rates exceed the coastal RSLR rate in San Francisco of 3.8 mm y^-1^ over our evaluation time period. Thus, the majority of stations also exceed the 2006-2018 global SLR rate of 3.7 mm yr^-1^, sometimes by a significant amount^1^. West of the Sacramento-San Joaquin confluence (i.e., Zone 1, Fig. 1), the highest RSLR rates are in the marshes of Grizzly Bay at Volanti Slough (7.6 mm y^-1^) and Goodyear Slough (12.9 mm y^-1^) (Fig. 1). Elsewhere in Zone 1, rates vary from 3.0 to 4.8 mm y^-1^. In the northern Delta (Zone 2, Fig. 1), the highest RSLR rate is 10.9 mm y^-1^ at Rio Vista. Rates are lowest around Sacramento (2.1–4.1 mm y^-1^), but are highly variable at other stations, ranging from 4.7 to 8.9 mm y^-1^. Finally, along the San Joaquin River and southern Delta (Zone 3, Fig. 1), the highest RSLR rates are at Venice Island (9.9 mm y^-1^), the Clifton Court intake for the State Water Project (10.1 mm y^-1^), and the Garwood Bridge near Stockton (10.9 mm y^-1^) (see Fig. 1 for locations). The lowest rates are at three stations along the Middle River (− 2.8, 0.5, and 2.5 mm yr^-1^; Fig. 3a). Elsewhere in the southern Delta, rates range from 3.2 to 7.9 mm y^-1^.

Our regression approach significantly improves upon RSLR rates calculated using a more traditional, conditional sampling approach in which river discharge effects are dealt with by considering only the dry, low river discharge season (June 1-November 30; see e.g., ref.^45^ for the annual hydrograph). After averaging this 6-month period for water years 2004–2022 and fitting a line, estimates of RSLR show more variability and uncertainty than our dynamics-based regression approach, even in San Francisco Bay. Our approach narrows the 95% confidence intervals of RSLR estimates by 89–99% (Fig. 3b; Supplementary Table S1). The linear fit RSLR estimates are also systematically lower along Delta channels, particularly along the Sacramento River. This bias occurs due to three years of relatively high discharge at the beginning of the period of analysis (water years 2004–2006) and three years of drought at the end (water years 2020–2022; Supplementary Figure S4a). This influence of discharge on water level and trends shows the necessity of an approach that accounts for both coastal and fluvial forcing to remove bias in RSLR estimates.

**Figure 5:**
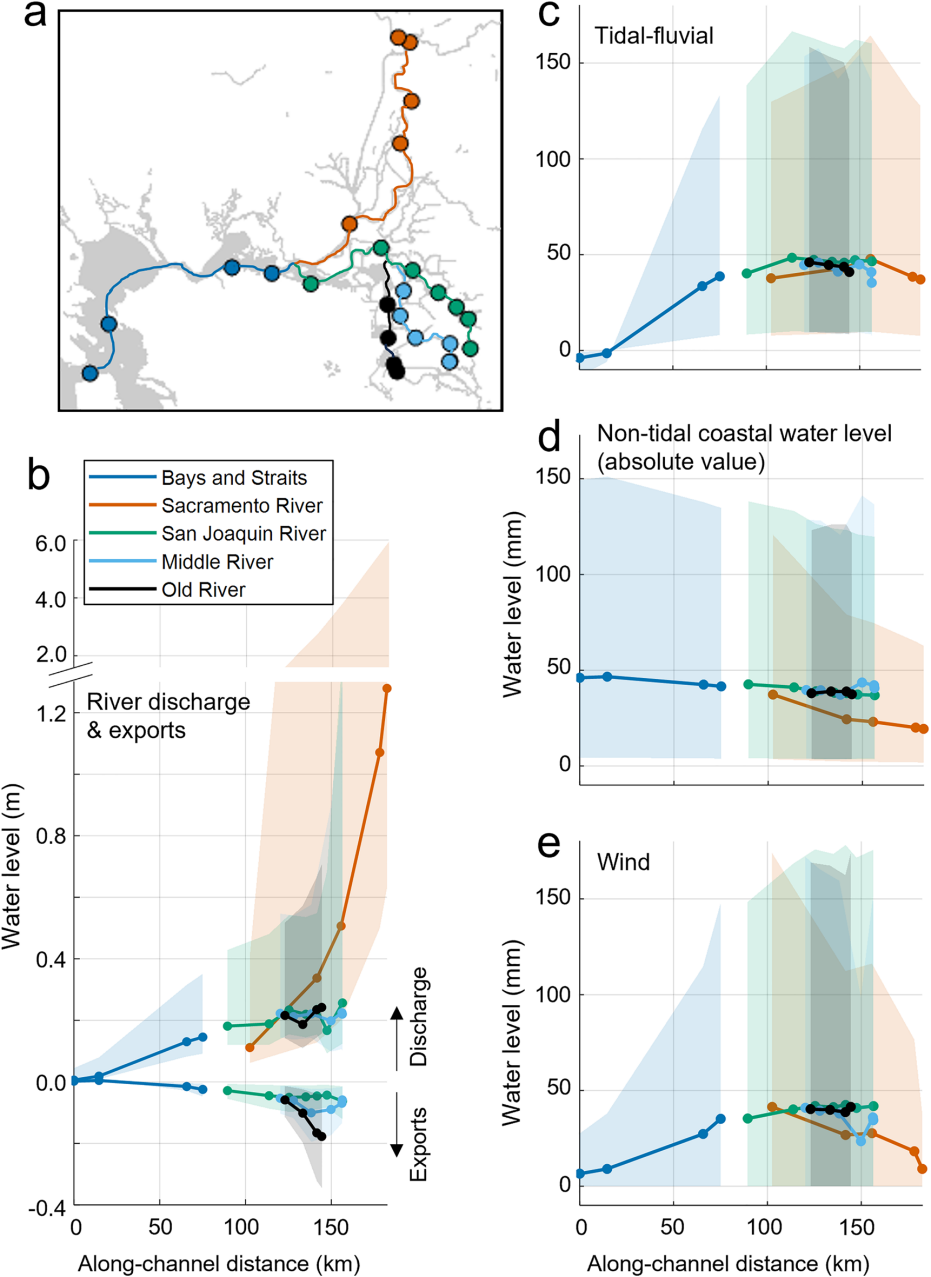
Physical drivers of daily water level in the Delta. (**a**) Map of the Delta showing gauge transects plotted in b-e along the bays and straits seaward of the Sacramento-San Joaquin Rivers confluence (blue), Sacramento River (red), San Joaquin River (green), Middle River (light blue), and Old River (black). (**b**–**e**) Spatially-varying influence of each regression term on water level for low, moderate, and high forcing conditions (Table 1). Along-channel distances are measured inland from the San Francisco gauge (westernmost blue dot in a). Lines show water level forcing for median values of river discharge, exports, tide range, the absolute value of non-tidal coastal water level (which has a symmetric distribution around 0), and wind speed. Shaded regions show forcing for the 25th to 75th quantiles. Note that for the tidal-fluvial term, the lower edge of the shaded region shows 25th-quantile tide range and 75th-quantile river discharge (minimizing Stokes drift), and the upper edge shows the reverse.

The super-elevation of DMWL in the Delta is strongly influenced by river flow, especially along the Sacramento River and during high flow conditions, but also includes a significant influence from wind, coastal sea-level perturbations, water diversions and pumping, and tidal-fluvial non-linear interactions (Figs. 4 and 5). Under median forcing (see Table 1), river discharge increases water levels by < 10 mm in San Francisco to ~ 1.3 m in Sacramento (QE term in Eq. 1; Figs. 4a, 5b). The 95th percentile river discharge (net delta outflow of 2500 m^3^ s^-1^) raises DMWL west of the Sacramento-San Joaquin confluence from 0.04 to 0.35 m (Zone 1, Fig. 1), in the southern Delta from 0.4 to 1.4 m (Zone 2; San Joaquin River flow of 420 m^3^ s^-1^), and in the northern Delta from 0.4 to 6 m (Zone 3; Sacramento River flow of 1,800 m^3^ s^-1^; Fig. 5b).

**Table 1:** Low, median, and high forcing conditions for physical process controlling daily mean water level in the Delta.

Forcing variable	Low forcing (5th percentile)	Median forcing (50th percentile)	High forcing (95th percentile)
Net delta outflow	170 m^3^ s^-1^	430 m^3^ s^-1^	2,500 m^3^ s^-1^
Sacramento river flow	200 m^3^ s^-1^	400 m^3^ s^-1^	1,800 m^3^ s^-1^
San Joaquin River flow	10 m^3^ s^-1^	50 m^3^ s^-1^	420 m^3^ s^-1^
Exports	− 40 m^3^ s^-1^	− 170 m^3^ s^-1^	− 330 m^3^ s^-1^
Tide range	1.2 m	1.7 m	2.5 m
Non-tidal coastal water level	± 0.005 m	± 0.05 m	± 0.18 m
Wind speed	0.1 m s^-1^	4.1 m s^-1^	8.4 m s^-1^

Water diversions in the southwestern region of the Delta (Fig. 1) lower DMWL. Median pumping rates (170 m^3^ s^-1^; Table 1) lower DMWL by up to 0.05 m along the San Joaquin River and nearly 0.2 m along the Old River (Figs. 4b, 5b). Elevated pumping during summer (95th percentile rate of 330 m^3^ s^-1^) lowers water level by up to 0.35 m in the southern Delta, with the greatest influence immediately adjacent to the water intakes and a negligible effect in the northern Delta (Fig. 4b, 5b).

Tidal influence on DMWL is prominent throughout the Delta on fortnightly (e.g., spring-neap) to seasonal time scales (a_5_ term in Eq. 1; Fig. 4c, 5c). The tidal-fluvial term in the regression captures the interacting influences of tidal range and river flow on DMWL (see Methods). Briefly, a greater river slope (the slope of the mean water level surface) is needed to discharge the same volume of water when the tidal range is larger. Thus, a large coastal tidal range causes greater setup at more inland stations. This effect is greatest when river discharge is low and diminished when discharge is high (see refs.^55,56^ for a detailed explanation). Results show that frictional tidal-fluvial interaction increasingly raises water level in the landward direction until ~ 160 km inland along the Sacramento River (near Freeport) and ~ 110 km inland along the San Joaquin, near the Old River confluence (Figs. 4c, 5c). When coastal great diurnal tidal range is high (95th percentile range of 2.5 m) and river discharge is low, these frictional effects raise DMWL by ~ 0.15 m in the Delta. High river discharge and small tidal ranges dramatically reduce the detected contribution of nonlinear tidal-fluvial interaction effects to < 20 mm throughout the Delta (Figs. 4c, 5c).

Non-tidal coastal perturbations in water level, caused for example by offshore wind forcing and storms, propagate far into the Delta, becoming small only in regions marked by significant fluvial forcing such as Sacramento (a_6_ term in Eq. 1; Fig. 4d). Daily averaged perturbations of ± 180 mm at Point Reyes (95th percentile; Table 1) raise or lower water level by 130–150 mm in the bays and straits west of the Delta Confluence (Zone 1), and 70–130 mm in northern and southern Delta channels (Fig. 5d). Thus, the daily coastal water level signal is attenuated by roughly 30–60%, depending on location. Non-tidal coastal water levels are shown as positive for illustrative purposes, but have a roughly symmetric distribution around zero (Supplementary Figure S4d).

Finally, wind setup from the predominantly westerly winds (Supplementary Figure S4f) increasingly raises DMWL inland from the Carquinez Straits, with 95th percentile wind speeds (8.4 m s^-1^; Table 1) raising water level 30–140 mm (a_7_ term in Eq. 1; Figs. 4e, 5e). Wind setup decreases landward along the Sacramento River and is spatially variable in the southern Delta, with 95th percentile wind speeds raising water level 80–170 mm along the Sacramento and 100–170 mm along southern Delta channels (Figs. 4e, 5e). The trends follow channel orientation (Fig. 5a), which increases fetch and wind setup along west-to-east trending channels and decreases or stays consistent along north–south trending channels (Fig. 5e).

## Discussion

### Relative sea level trends

Relative sea-level rise effects captured by the model are spatially heterogeneous (Fig. 3, Supplementary Table S1); subsidence inferred by satellite-based VLM measurements of Delta Islands are similarly variable^59,60^. The correlation between daily averaged sea-level fluctuations at the coast and in the Delta (Fig. 4d) also shows that coastal perturbations propagate far into the Delta with a relatively small attenuation, except in the fluvial Sacramento River (Figs. 4d and 5d). Thus, oceanic SLR, which occurs over much longer time scales, is also a major, statistically significant driver of RSLR in the Delta. Based on this consideration, regression-based RSLR estimates at Delta tide gauges minus an oceanic SLR rate estimated at San Francisco (corrected for VLM) yield an approximate estimate of subsidence rates at Delta gauges.

Over the 2004–2022 period, the RSLR rate at the San Francisco gauge was 3.8 ± 0.2 mm yr^-1^ (Supplementary Table S1). Present VLM estimates based on two Global Navigation Satellite System (GNSS) instruments within 10 km of the San Francisco gauge suggest a coastal VLM rate of –0.5 ± 0.5 mm yr^-1^ (GNSS gauge TIBB and UCSF; data processing from the Nevada Geodetic Laboratory, using methods described in ref.^61^). Using this VLM estimate yields a local oceanic SLR rate of about 3.3 ± 0.6 mm yr^-1^, which is similar to the 1993–2018 global SLR rate^1^. The significant variability in Delta RSLR rates relative to the coastal rate of 3.3 mm yr^-1^ indicates that local/regional factors play a substantial role. Subtracting coastal SLR from Delta RSLR rates yields VLM rate estimates at gauges west of the Sacramento-San Joaquin confluence of − 9.6 to + 0.3 mm yr^-1^ (Zone 1; median − 0.5 mm yr^-1^), in the northern Delta of − 7.6 to + 1.2 mm yr^-1^ (Zone 2; median − 3.5 mm yr^-1^), and in the southern Delta of − 7.6 to + 6.1 mm yr^-1^ (Zone 3; median − 2.4 mm yr^-1^).

These tide gauge-based VLM estimates are lower than the regional mean -of − 9.2 ± 4.4 mm yr^-1^ VLM rate inferred by satellite measurements^59^, except for at six stations: Goodyear Slough in Grizzly Bay, Rio Vista along the Sacramento River, Venice Island, the Clifton Court intake for the State Water Project, and the Garwood Bridge near Stockton in the southern Delta (Fig. 3a). The satellite-based mean estimate primarily captures VLM of Delta islands, where it has been hypothesized that soil oxidation is the dominant driver of subsidence^40^. Soil oxidation does not affect the riverbed and, in turn, should not affect tide gauges mounted on piers or alluvial levees. Thus, our median VLM estimate of − 2.5 mm yr^-1^ for the Delta (Zones 2 and 3 in Fig. 1), while still large, is substantially less than land-surface subsidence due to oxidation. Uncertainty in our VLM estimates is influenced by regression-based RSLR uncertainty (up to ± 0.5 mm yr^-1^), tide gauge leveling history and data quality, and the lack of a co-located GNSS measurement at the San Francisco gauge. GNSS-based VLM estimates also have shifted over time for San Francisco, as they still depend on the record length, geoid solution, and processing method^61^.

Our estimats of VLM recorded at tide gauges are likely driven in part by consolidation of soils from groundwater variations, which were modeled to cause 30–40% of surface subsidence in some western Delta Islands^40^. Natural gas extraction also occurs at the periphery of the Delta, and the largest natural gas field in California is centered in the northwestern Delta near Rio Vista (Site 3 in Fig. 1; see e.g., refs.^48,62^). Based on estimates from sediment cores in a western Delta wetland (closest station: Site 5, Fig. 1), deep VLM of up to -3 to -5 mm yr^-1^ occurred between 1963 and 1989, probably tapering off as natural gas production decreased^48,61^. Thus, gas extraction likely contributes to the anomalously high RSLR rates in Rio Vista (10.9 ± 0.3 mm yr^-1^) and nearby locations (e.g., sites 4 and 5 in Fig. 1; see Supplementary Table S1 for rates). Other broad-scale contributions to VLM include glacial isostatic adjustment (about − 1 mm yr^-1^ in the San Francisco region; ref.^63^) and plate tectonics, which is causing some uplift in the southwestern Delta and eastern San Francisco Bay Area^64^. Kilometer-scale variations in our VLM estimates may be explained by processes associated with near-surface consolidation, rather than regional-scale groundwater variations, gas extraction, glacial isostatic adjustment, or tectonics. We do not attempt to identify specific causes of VLM variability, as this requires specialized field methods and models^40,62^. Rather, our aim is to estimate RSLR variability using water levels, a data source that provides direct measurements of RSLR, but that has not often been used here or in other Deltas.

Results also show that DMWL in the Delta is substantially influenced by river flow, tides, water diversions/extractions, and wind (Figs. 4 and 5); hence, processes that change the response of water level to these forcing factors could alter mean water levels and RSLR, potentially challenging the assumption of stationarity underlying our statistical model. Historically, it is likely that large-scale changes to river depth, such as dredging of the Stockton shipping channel in the 1930s from ~ 3 to ~ 8 m, lowered mean water levels; this has been observed in other locations^9,10,52,65^. Deeper water reduces the river slope required to push river discharge toward the ocean, and wind stress is less effective at raising deep than shallow water. Deepening from RSLR, if sedimentation does not adjust depths, can also alter the propagation of coastal water level perturbations into a tidal river^65–67^. Major deepening projects have not occurred over the past two decades, and if there are stations where geomorphically-caused changes in river flow and wind setup changed mean water level, they were likely removed during the quality-assurance process (see Methods). Thus, the assumption of stationarity underlying our statistical model is reasonable, but model use further back in time might require a piecewise calibration approach to different decades (see e.g., refs.^44,52^). We note that in general, each meter of future SLR at the coast may produce less setup far up-river, with the decrease in setup also depending on river flow^65–67^; thus, hydrodynamic modeling may be required to project future SLR and evaluate the barotropic response. Similarly, it remains unclear whether VLM rates estimated here will remain constant. Additional insights regarding system responses may be gained from retrospective studies that evaluate the many archival water level records from the Delta^68,69^.

Rapid and spatially variable RSLR rates driven by VLM, as also found by other methodologies^42,59^, pose a challenge to flood prevention efforts and climate change adaptation within the Delta region. The spatial variation in RSLR observed at gauges (this study) is likely also present as smaller scales, as lidar-based estimates of levee subsidence suggest > 10 mm yr^-1^ in variation of VLM rates over spatial scales of less than 500–1000 m^42^. Moreover, spatially variable RSLR interacts with a variable sensitivity to river flow, wind, and coastal forcing (Figs. 4 and 5). Thus, assessment of future flood hazard in the Sacramento-San Joaquin Delta and other delta regions impacted by VLM requires projections of hydro-meteorological changes, sea-level changes, the resulting hydrodynamic response, and hyperlocal consideration of shoreline VLM.

### Daily water level variability

Results show that water levels in the Delta region (Fig. 1) are in a transition zone between fluvial and coastal dominance, but are also strongly influenced by wind and water resources management. Upstream of Rio Vista on the Sacramento River, variations in DMWL are driven by river discharge. In other regions, wind effects, tidal-fluvial interaction, non-tidal coastal water level perturbations, and extractions are of the same order of magnitude as median river discharge effects, and can be significant contributors to water level even during high river discharge. Perturbations in DMWL driven by non-tidal coastal water level variability are typically 30–60% of their coastal value. This suggests that coastal SLR occurring over decadal time scales influences the entire region and will become more prominent as coastal SLR rates accelerate^1^. However, our model results also suggest that any potential future decreases in mean river flow^32^ may suppress mean water levels in a location-dependent manner, particularly on the Sacramento River. Over secular time scales, reduced river flow^44^ has probably also decreased water levels. Similarly, future plans to reduce pumping in the southern Delta and instead divert up to 170 m^3^/s of water around the eastern Delta^70^, will likely decrease Sacramento River discharge, lower water levels in the northern Delta, and raise them in the southern Delta.

Both hydrological trends and water resources management can influence mean sea-level (and potentially trends) in tidally influenced regions far from the coast (see also refs.^9,10^). Though our approach only evaluates daily mean water levels, experience in tidal rivers suggests that tidal datums and extremes will also shift (see e.g., ref.^52^). Thus, understanding the factors that influence base-water levels provides insights into the evolution of extreme water levels and hazards, such as the increased future probability of a megaflood^46^.

### Model applications and limitations

Our approach (Eq. 1) enables a simple estimation of spatially varying impacts of physical forcing on DMWL. Unlike numerical models, a statistical model runs quickly and does not require detailed bathymetry. Our approach can therefore be applied to other delta and tidal river regions to quantify RSLR and evaluate the impacts of river, coastal, and human forcing on mean water levels. The statistical model can also be applied to “what-if” analyses that infer the spatially varying effects of future water diversions or changes in water supply on mean water levels and RSLR. Because we focus here on mean water levels, our regression model is not optimized for high flow, flood conditions; nonetheless, the regression could be further developed to estimate high and low waters in addition to mean water levels^52,54,71^. Combined with an evaluation of levee heights^42^ or wetland flora elevations, such an analysis should be able to evaluate future flood risk from compound riverine-coastal events or the future zonation of wetlands (for which Mean High Water is an important boundary).

Our regression approach also has limitations. As with all instrumental tide gauge records, undocumented adjustments in the gauge zero or datum may compromise individual records. Our quality assurance, which included examining residual time series for unexplained jumps, minimizes but may not completely remove such issues. In this context, however, low residual errors and consistent spatial patterns in water level forcing (e.g., water level variability from river flow and wind) among the large number of gauges within the Delta bolsters our conclusions. We also do not include all potentially relevant water level forcings in the regression. Attempts to add additional regressors, such as additional inflows from the Consumnes and Mokelumne Rivers, two minor tributaries, did not improve model results. We also did not consider seasonally constructed barriers in the southern Delta (e.g., Old River, Middle River, Grant Line Canal), which are known to affect local water level slopes and flow routing^53^. Some stations adjacent to barriers were removed by initial quality control (see Methods) or due to poor regression error statistics (Station 35 upstream of an Old River barrier; Supplementary Fig. S3). For the remaining barrier-adjacent stations in the southern Delta, temporary channel closures may explain the relatively large RMSE values and anomalously low RSLR rates (Figs. 2a and 3). Additional tools such as wavelet tidal analysis^72^ and numerical models may be needed to assess these local water level effects.

Similarly, we were unable to improve the regression by accounting for the flow-dependent routing of water. In a delta, the division of tidal and river flows through a channel network changes as a function of both river flow and tides^11,73^. In the Sacramento-San Joaquin Delta, water is diverted from the northern to the southern Delta through the Cross Delta Channel at low flows, while at high flows, water is rerouted through floodplains such as the Yolo Bypass (see Fig. 1 for locations). Initial attempts to account for this variability were unsuccessful. Given that accurate prediction of high and low waters is not the purpose of this analysis, we omit the 1% of days where net Delta outflow exceeds the 99th percentile and disregard results at stations displaying high residual errors at low flows. For a more complex analysis, an idealized tidal network model^66^ or a numerical model may be required.

Our approach also excludes any time lags between forcing and the DMWL response. For example, the DWL response to Sacramento River discharge on a given day is modeled based on average discharge over the same 24-h period throughout the Delta when, in actuality, the response at distal gauges along the San Joaquin River likely lag the response of nearby Sacramento River gauges. Inclusion of spatially varying time lags should be investigated if the water level forecasts or hindcasts need to be optimized. Here, we sought to impose a uniform approach for all stations, to provide comparability between stations and obtain first order insights into the fluvial and coastal factors which influence water levels.

## Conclusions

We adapt a regression model developed by Refs.^52,54–56^ to estimate spatially varying rates of RSLR and controls on daily mean water level at 50 stations in the Sacramento-San Joaquin Delta region over water years 2004–2022. The regression yields quantitative estimates of daily water level variability driven by coastal, hydrological, and meteorological factors. Under typical (median) forcing conditions, river discharge raises water level by 0–1.3 m, depending on location, and water diversions in the southwestern Delta suppress water levels by up to 0.2 m. During elevated discharge and pumping (95th percentile), water levels are raised up to 6 m and lowered by up to 0.35 m. Daily water level variations at the coast are attenuated by 30–60%, depending on location, and strong westerly winds (95th percentile) raise water levels by 0.03–0.17 m. Finally, tidal-fluvial interactions increase daily mean water level up to 0.05 m during median tidal range and discharge conditions, and up to 0.15 m when tidal range is large and discharge small. Clearly, our regression model produces quantitative insights into the major factors that cause water level to vary, and that can bias RSLR estimates. Moreover, the methods developed here can be applied to improve RSLR estimates in deltas and tidal rivers around the world.

Regression-based RSLR estimates range from − 2.8 to 12.9 mm yr^-1^, with VLM driving significant variability over the 3000 km^2^ Delta region. Compared with RSLR estimates based on a linear fit to dry season water levels, our dynamically-based nonlinear regression approach narrows the 95% confidence interval by 89–99% and removes a low bias driven by recent drought. Based on oceanic SLR in San Francisco, we estimate that VLM rates at the 41 Delta stations evaluated in Zones 2 and 3 (Fig. 1) range from − 9.6 to + 6.1 mm yr^-1^, with a median of − 2.5 mm yr^-1^. The large VLM recorded by Delta gauges may be driven by soil consolidation, groundwater extraction, and/or deep subsidence caused by natural gas extraction; other factors include glacial isostatic adjustment and plate tectonics.

The river network within the Delta contains population centers, agricultural resources, wetland habitat, and critical water conveyance infrastructure. Thus, spatially heterogenous Delta RSLR implies that sub-regions will experience significantly different sea-level rise story lines in the future as global SLR accelerates, with implications for ecological adaptation, the evolution of flood risk at levees, and infrastructure planning. This conclusion is generalizable to other deltaic regions that are densely settled and known to be impacted by large and spatiotemporally variable subsidence, such as Shanghai, Jakarta, Tokyo, and New Orleans.

## Methods

### Daily water level regression

We model daily mean water level (DMWL) at 50 stations in the Delta region as a function of forcing by river flow, tides, non-tidal coastal sea level, wind, water diversions (pumping exports), and long-term relative sea-level change. We adapt a modeling approach developed by Refs.^52,54–56^ and based on the theory of tidal propagation and mean water levels in a convergent channel with friction and river forcing^74^. While the original version of this regression model considered only tidal-fluvial interactions, the model has been augmented by adding additional forcing (coastal factors, multiple flow sources) and broadened by allowing exponents to be set iteratively within ranges based on theory. While the model is somewhat heuristic, it is based in theory and well tested [e.g.,^52,54,65^]. In this contribution, we further develop the model by adding terms for pumping (export), wind, and sea-level trends, and we represent coastal forcing using a non-river flow influenced tidal gauge (see below).

We first explain the regression used to model DMWL, and we describe the forcing data in subsequent sections. At each tide gauge, we employ the following regression:

1 $$\begin{array}{*{20}c} {DMWL = a_{0k} + QE\left( b \right) + a_{5k} T^{n2} Q_{N}^{ - n3} + a_{6k} C + a_{7k} W^{2} + a_{8k} t,} \\ \end{array}$$

$$QE\left( b \right) = \left\{ {\begin{array}{*{20}l} {a_{1k} \left( {Q_{N} - E} \right)^{n1n} + a_{4k} E, \;\;\;\;b = 1} \hfill \\ {a_{2k} Q_{S}^{n1s} ,\;\;\;\; b = 2} \hfill \\ {a_{2k} Q_{S}^{n1s} + a_{3k} \left( {Q_{J} } \right)^{n1j} + a_{4k} E, \;\;\;\;b = 3} \hfill \\ \end{array} } \right.$$

where (see Fig. 1 for all locations described below):

*DMWL*: Daily mean water level (m)
*QE(b)*: Discharge/exports term, dependent on geographic zone *b*. Note that *E* is negative.
*Q*_*N*_: Daily net delta outflow index (NDOI; m^3^ s^-1^), estimated at Chipps Island
*E*: Combined daily exports from the Central Valley Project and State Water Project (m^3^ s^-1^). Note that *E* is negative.
*Q*_*S*_: Daily Sacramento River flow at Freeport (m^3^ s^-1^)
*Q*_*J*_: Daily San Joaquin River flow at Vernalis (m^3^ s^-1^)
*T*: Daily great diurnal tidal range (m), or HHW- LLW at Point Reyes.
*C*: Daily non-tidal coastal sea level at Point Reyes (m)
*W*: Daily mean wind speed (magnitude) at Port Chicago (m s^-1^)
*t*: Time (days)
*a*_*0k*_–*a*_*8k*_: Regression coefficients (unique for each station)
*k*: Water level station index
*n*_*1n*_* n*_*1s*_* n*_*1j*_: River flow exponents (unique for each station)
*n*_*2*_* n*_*3*_: Tidal-fluvial interaction exponents (unique for each station)
*b*: Geographic zone of each station

The *a*_0_ term on the right-hand side of the equation relates DMWL to a local datum. Term *QE(b)* represents the effects of river discharge and exports (via pumping) on DMWL and varies depending on the geographic zone a station is located in (zones 1–3, Fig. 1). In the western bays and straits (zone 1, west of the Sacramento-San Joaquin Rivers confluence), DMWL is affected by net Delta outflow (*Q*_*NDOI*_*—E*, see the *River Discharge and Exports* section below for an explanation of this term) and pumping in the southwestern Delta (*E*). In the northern Delta (zone 2), Sacramento River discharge influences DMWL (*Q*_*S*_), while effects of distal pumping in the southern Delta and San Joaquin River flows are negligible and not included. In the southern Delta (zone 3), San Joaquin River discharge (*Q*_*J*_), Sacramento River discharge (via backwater effects), and pumping all influence DMWL. The decision regarding discharge terms to include or exclude was made through experimentation.

The *a*_*5*_ term represents the interacting influences of variations in tidal range (generating more friction; often called spring-neap variability) and river flow on DMWL. As explained in the Results section, larger tides produce more frictional drag, which is balanced by a steeper surface slope in the river discharge. This steeper slope results in a greater setup at more inland locations. The effects are greatest when river discharge is low and diminished when discharge is high (see refs.^55,56^ for a detailed explanation). Following Ref.^52^, and based on experimentation, we use the great diurnal tidal range (GDTR) as the proxy for tidal forcing; effectively, this means we are assuming that the largest daily tides have the greatest effect, due to friction being nonlinear.

The *a*_*6*_ term represents non-tidal coastal sea level variation driven, for example, by winds, pressure, steric effects, and upwelling. Specifically, coastal sea level in this region is influenced by the northwesterly winds during the spring and summer that drive seasonal upwelling and lower sea level along California’s coast^75^. Past investigations have demonstrated that low-frequency, seasonal sea level changes in San Franciso Bay are primarily controlled by coastal sea level variations such as upwelling^76,77^. This regression term also accounts for the inverted barometer effect, as the spatial scale of high- and low-pressure systems is typically much larger than that of the Delta region. The *a*_*7*_ term represents local (estuary-scale) wind forcing that drives setup in the Delta relative to the coastal ocean. Wind speed is squared, to represent the well-known proportionality between surface stress and wind speed. Finally, the *a*_*8*_ term represents the long-term secular trend in DMWL.

Model exponents have theoretical values of *n*_*1n*_*, n*_*1s*_*, n*_*1j*_ = 2/3; *n*_*2*_ = 2; and *n*_*3*_ = 4/3^55^, but have been allowed to vary over plausible ranges of 0.5 ≤ *n*_*1n*_*, n*_*1s*_*, n*_*1j*_ ≤ 2; *n*_*2*_ ≤ 2; and 0.5 ≤ *n*_*3*_ ≤ 1.6^54^. At each site, we iteratively test combinations of exponents in these ranges and choose the set of values that minimizes RMS error. Coefficients *a*_*0k*_–*a*_*8k*_ are determined by robust multiple linear regression using the *robustfit* MATLAB function to fit the right-hand side of Eq. 1 to measured DMWL at each station. Robust linear regression iteratively downweights outliers, reducing their influence on model fit.

We evaluate regression model skill using the adjusted squared error (R^2^), the root mean square (RMS) residual error, and by assessing whether residuals (modeled minus measured DMWL) are correlated with forcing parameters (discharge, tidal range, etc.). Model skill was affected by water-resource management, which employs a system of tide gates, weirs, and seasonal barriers that routes water differently at high and low river discharges. High residuals on days of low or extreme high river discharge therefore likely indicate the presence of management effects that may bias the RSLR estimate. Indeed, including flood periods yielded large and biased residuals at most stations in the northern and southern Delta (Supplementary Figs. S2, S3). Given that accurate prediction of high waters is not the purpose of this analysis, we improve model performance by omitting the 1% of days where net Delta outflow exceeds the 99th percentile of 5640 m^3^ s^-1^.

### Water level data, datum adjustments and quality control of tide gauge data

Multiple agencies measure water levels in the Delta region. The National Oceanographic and Atmospheric Administration (NOAA) maintains two gauges near the boundary of San Francisco Bay and the Delta: Port Chicago (1973-present) and Martinez (2013-present). More than 100 additional Delta water level datasets are available from the California Data Exchange Center (CDEC; https://cdec.water.ca.gov/) at hourly resolution, and from the GESLA-3 data set^78^. These data are collected by agencies including the US Geological Survey (USGS), the California Department of Water Resources (CA-DWR), and the US Bureau of Reclamation. Measurements from the CA-DWR are also available at the California Water Data Library (CA-WDL; https://wdl.water.ca.gov/). Some records start as early as 1982, though most start in the 1990s or 2000s. High-quality records with a known datum are available from USGS starting in 2007, and offsets for a change to the NAVD-88 datum in 2005 and the conversion from the earlier datum (NGVD-29) are available from CA-DWR. Many periods of high-quality data exist before 2007, but quality control indicates there are periods with uncertain time stamps and datum shifts in some of the records.

For this effort, we concentrate on a subset of 50 gauges with near-complete records over water years 2004–2022 (October 1, 2003 to September 30, 2022). Data were quality assured using an algorithm from Ref.^79^ that identifies and removes: (1) erroneous interpolations of statistic water levels (e.g., no change > 4 h); (2) outliers based on the local median absolute deviation (i.e., Hampel filter); and (3) data with unusually high variability at high river flow conditions, when tidal friction otherwise attenuates tides^7^. Additionally, the algorithm used a buddy gauge approach to identify undocumented datum shifts in the data^80^. This quality control process may have eliminated stations where events such as drought barrier installations, habitat restoration projects, or levee breaks occurring between 2004 and 2022 led to gradual or sudden changes in mean water level.

### Daily mean water level

Daily mean water level calculated strictly as the mean water level over a 24-h period would be biased by tides, as 24 h is not an exact multiple of the tidal period. We therefore apply a 45-h Lanczos filter to remove tidal variation and then take the water level at 12:00 each day as the daily water level input for the regression. A 45-h filter length was chosen using a sensitivity test showing the high-passed variance minimally increased for window sizes > 45 h.

### River discharge and exports

Daily discharge from three locations is input to the regression: Net Delta Outflow Index (NDOI), Sacramento River, and San Joaquin River (Fig. 1, Supplementary Fig. S4a). NDOI is an estimate of net delta outflow at the Sacramento-San Joaquin rivers confluence produced by CA-DWR’s Dayflow model (https://data.cnra.ca.gov/dataset/dayflow). Dayflow estimates net daily Delta outflow by evaluating river inflow from Delta tributaries and estimating losses due to pumping, evaporation, and other miscellaneous withdrawals and diversions. NDOI does not consider fortnightly water storage effects caused by tidal Stokes drift and nonlinear friction^45^. Note that for the geographic zone 1 (*b* = 1) *a*_*1*_ river discharge term, we add exports back into NDOI (written as *NDOI – E* in Eq. 1 because *E* is negative) because exports are already accounted for with the *a*_*4*_ export term. For the other two river inputs, we use river discharge estimates from the USGS at the Sacramento River at Freeport (station 11447650) and the San Joaquin River near Vernalis (station 11303500). Daily mean values are retrieved from the published Dayflow results.

Dayflow results also include pumping rates for the Central Valley Project at Tracy; the Contra Costa Water District Diversions at Middle River, Rock Slough, and Old River; the State Water Project at Banks Pumping Plant and the Clifton Court Intake; and the North Bay Aqueduct (Fig. 1, Supplementary Fig. S4b). We exclude exports from the North Bay Aqueduct because they are small (maximum 6 m^3^ s^-1^ over water years 2004–2022) and sum exports from the three southern Delta water projects into a single forcing term (Dayflow variables QCVP + QCCC + QSWP). This is reasonable because temporal variability in pumping rates is similar for the three projects, QCCC is small relative to QCVP and QSWP, and the intakes for QCVP and QSWP are close to each other and likely produce an overlapping response in Delta water levels.

All regression calculations are made in UTC, and Dayflow outputs daily mean values calculated based on local time. We therefore interpolate daily flows and exports to hourly time series, convert to UTC time, then recalculate daily means.

### Tidal range and non-tidal coastal water level

We force the regression with tide range and non-tidal coastal water level at the NOAA Point Reyes tide gauge (station 9415020), which is located on the Pacific coast ~ 50 km northwest of San Francisco (Fig. 1, Supplementary Fig. S4c-d). We first detrend hourly Point Reyes water levels by fitting a linear trend to monthly mean water level from 2004 to 2022, then subtract the calculated trend from hourly data. We calculate hourly predicted water level for the detrended Point Reyes time series using the MATLAB-based harmonic analysis program UTide^81^. Tidal constituents and predictions are independently estimated for each year from a 369-day analysis^82^. We exclude annual and semiannual constituents SA and SSA because they are generally not a result of astronomical forcing and constituents MM and MF because they often have large uncertainty in harmonic analysis^83^. The daily great diurnal range is calculated by differencing the predicted Higher-High Water and Lower-Low Water each day.

We calculate daily mean non-tidal coastal water level by subtracting predicted hourly water level from detrended hourly measured water level, then calculating the mean over each 24-h period. The resulting daily data set includes coastal water level perturbations on many time scales, including storm surge, seasonal upwelling and downwelling, and interannual variations in sea-level (e.g., caused by El Nino).

### Wind

We force the regression with wind magnitudes from the NOAA Port Chicago station (station 9415144; Fig. 1, Supplementary S4e). We chose this station because of: (1) its high data quality; (2) the long westerly fetch from Suisun Bay to the Sacramento-San Joaquin Rivers confluence that is aligned with the region’s predominantly westerly winds (Supplementary Fig. S4f); and (3) the location of the anemometer on a pier, which suggests that it primarily measures wind over water, not over land. We calculate daily mean wind speed (the mean over each 24-h period) from hourly wind magnitudes. We only include a wind magnitude measurement if the associated azimuth is from the SE, S, SW, W, and NW (i.e. we omit winds with azimuths from 315º through 135º) because winds that move water northward and eastward through the bays and straits entering the Delta are those most likely to drive setup. Winds from the opposite direction could drive set down, but they only account for 18% of wind at Port Chicago, where winds primarily come from the WSW (Supplementary Fig. S4f).

### Supplementary Information

Supplementary Figures.

Supplementary Table S1.

## Data Availability

Datasets and code for running the regression are available in the HydroShare repository, https://www.hydroshare.org/resource/60a77d2b6df446d8bb4390cd94712459. Code for quality-assuring tide gauge data is available in the HydroShare repository, http://www.hydroshare.org/resource/47505deb95cc4df08a9de4d1e1641f71.
