# Supplementary material for: Sea level rise and the drivers of daily water levels in the Sacramento-San Joaquin Delta
Source: Sci Rep. 2023 Dec 17;13:22454. doi: 10.1038/s41598-023-49204-z (PMC10725870; doi:10.1038/s41598-023-49204-z)
Supplement: Supplementary file 1 — Supplementary Figures. [file 41598_2023_49204_MOESM1_ESM.docx]

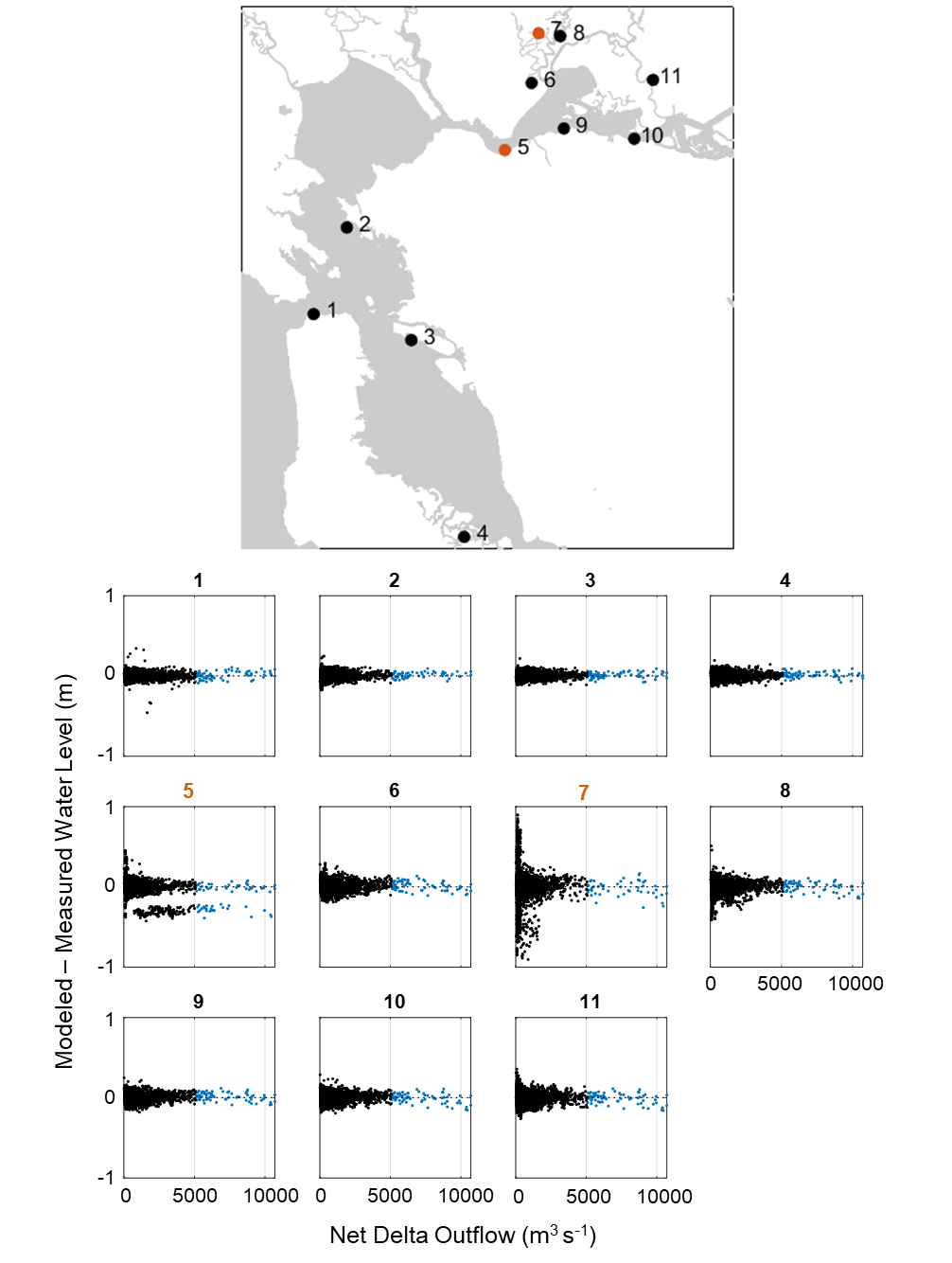


**Supplementary Figure S1.** Relationship between river discharge and residual error in bays and straits west of the Sacramento-San Joaquin Rivers confluence. The 1% of days excluded from the final regression analysis where Net Delta Outflow exceeded the 99^th^ percentile are plotted in blue. Regression results at stations 5 and 7 (shown in red) are not considered reliable due to large residuals that are likely related to management effects (station 7) or undocumented datum shifts (station 5).


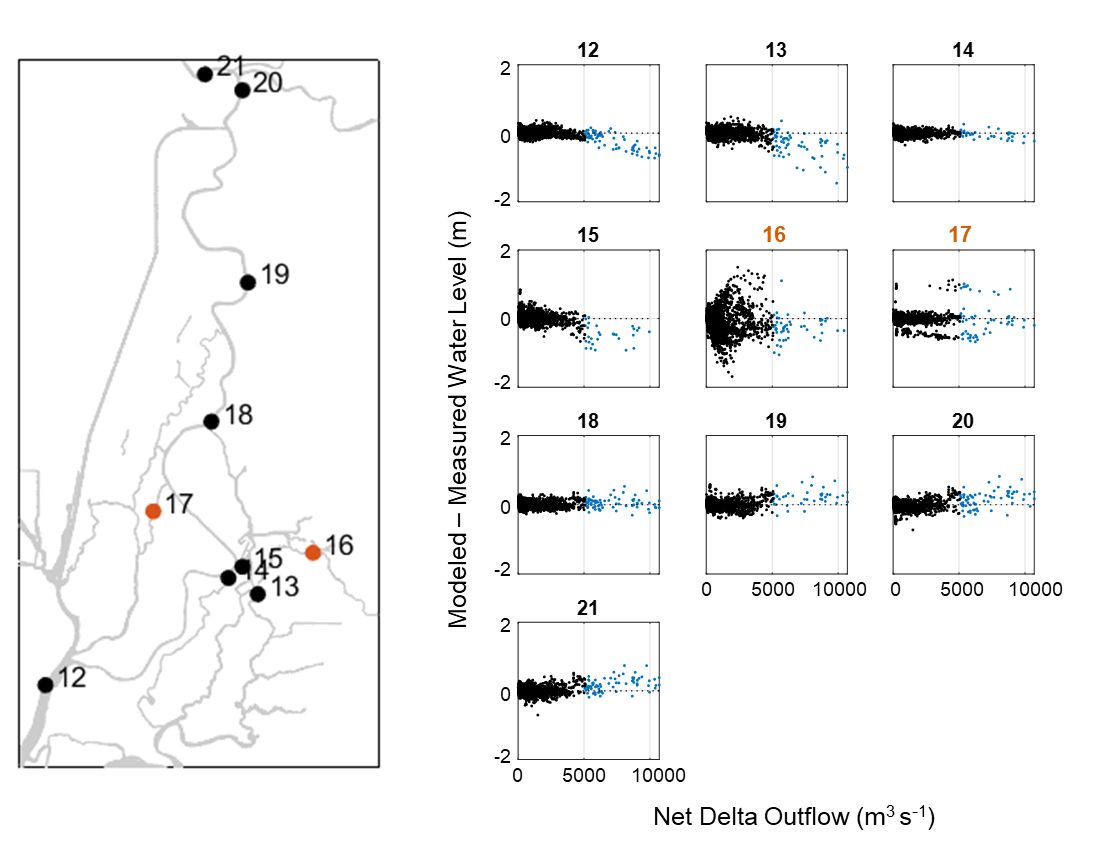


**Supplementary Figure S2.** Relationship between river discharge and residual error in the northern Delta. Regression results at stations 16 and 17 (shown in red) are not considered reliable due to large residuals that are likely related to inflows not captured by the regression (station 16) or undocumented datum shifts (station 17).


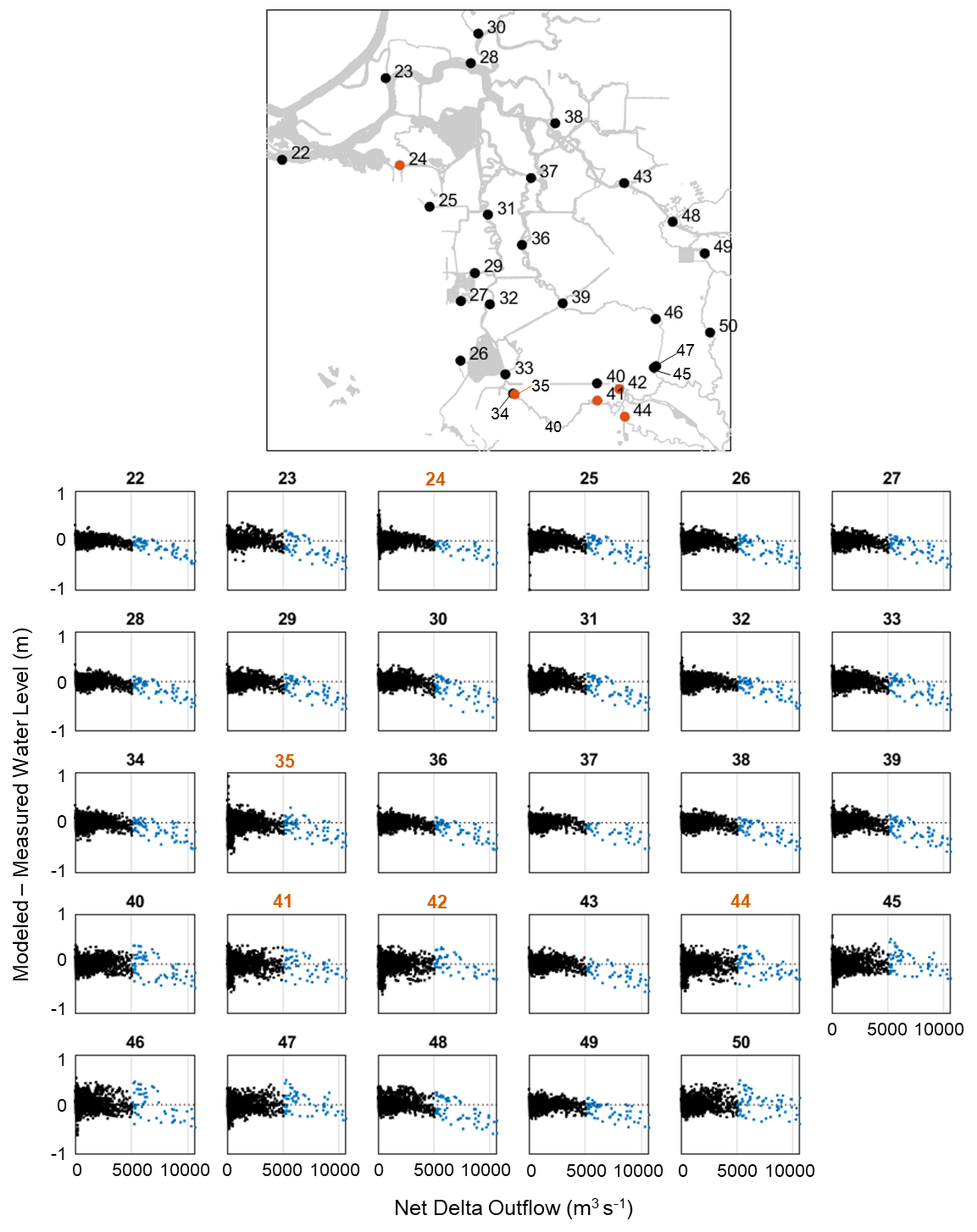


**Supplementary Figure S3.** Relationship between river discharge and residual error in the southern Delta. Regression results at stations 24, 35, 41, 42, and 44 (shown in red) are not considered reliable due to large residuals that are likely related to inflows not captured by the regression or management.


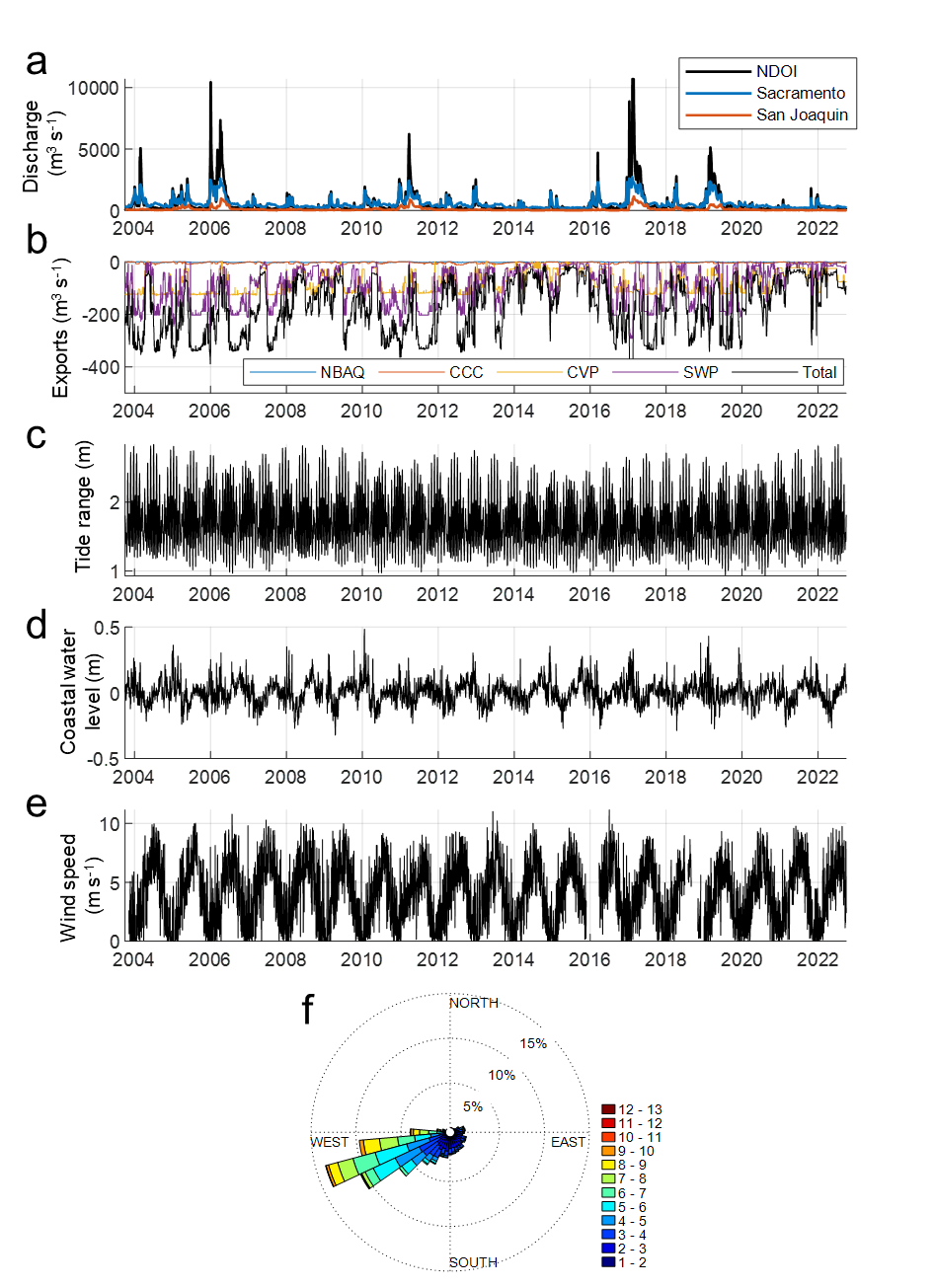


**Supplementary Figure S4.** Time series of daily water level forcings for water years 2004-2022 used as regression inputs. a) Daily mean river discharge at three locations: net delta outflow at Chipps Island (NDOI), Sacramento River at Freeport (USGS station 11447650), and San Joaquin River near Vernalis (USGS station 11303500). b) Daily exports (pumping rates) for the North Bay Aqueduct (NBAQ; not used in regression), Contra Costa Water District Diversions (CCC), the Central Valley Project (CVP), and the State Water Project (SWP). Total = CCC + CVP + SWP and is the final input into the regression. Values are from published Dayflow results. b) Daily greater diurnal tide range (higher high water minus lower low water) at Point Reyes (NOAA station 9415020). c) Daily mean non-tidal coastal water level (detrended and detided) at Point Reyes. d) Daily mean wind speed at Port Chicago, only including winds from the SE, S, SW, W, and NW (NOAA station 9415144). e) Wind rose diagram for Port Chicago, showing prevailing winds from the WSW.
